# Ultrafast atomic-scale visualization of acoustic phonons generated by optically excited quantum dots

**DOI:** 10.1063/1.4998009

**Published:** 2017-08-07

**Authors:** Giovanni M. Vanacore, Jianbo Hu, Wenxi Liang, Sergio Bietti, Stefano Sanguinetti, Fabrizio Carbone, Ahmed H. Zewail

**Affiliations:** 1 Physical Biology Center for Ultrafast Science and Technology, Arthur Amos Noyes Laboratory of Chemical Physics, California Institute of Technology, Pasadena, California 91125, USA; 2 Institute of Physics, Laboratory for Ultrafast Microscopy and Electron Scattering (LUMES), École Polytechnique Fédérale de Lausanne (EPFL), Lausanne, Switzerland; 3 Laboratory for Shock Wave and Detonation Physics Research, Institute of Fluid Physics, China Academy of Engineering Physics, Mianyang, Sichuan 621900, China; 4 Wuhan National Laboratory for Optoelectronics, Huazhong University of Science and Technology, 1037 Luoyu Road, Wuhan 430074, China; 5 L-NESS and Dipartimento di Scienza dei Materiali, Università di Milano Bicocca, Via Cozzi 53, I-20125 Milano, Italy

## Abstract

Understanding the dynamics of atomic vibrations confined in quasi-zero dimensional systems is crucial from both a fundamental point-of-view and a technological perspective. Using ultrafast electron diffraction, we monitored the lattice dynamics of GaAs quantum dots—grown by Droplet Epitaxy on AlGaAs—with sub-picosecond and sub-picometer resolutions. An ultrafast laser pulse nearly resonantly excites a confined exciton, which efficiently couples to high-energy acoustic phonons through the deformation potential mechanism. The transient behavior of the measured diffraction pattern reveals the nonequilibrium phonon dynamics both within the dots and in the region surrounding them. The experimental results are interpreted within the theoretical framework of a non-Markovian decoherence, according to which the optical excitation creates a localized polaron within the dot and a travelling phonon wavepacket that leaves the dot at the speed of sound. These findings indicate that integration of a phononic emitter in opto-electronic devices based on quantum dots for controlled communication processes can be fundamentally feasible.

## INTRODUCTION

The understanding and active control of quantum materials are crucial aspects for addressing the technological challenges of the 21st century, mainly associated with the pressing demands for sustainable energy, high-speed communication and high-capacity data storage. In recent years, low-dimensional materials have shown great promises to satisfy these demands due to their unique electronic and structural properties. When reaching dimensions of few unit cells, spatial confinement of electrons, photons, and phonons results in the formation of multiple quantum states, whose competition and stabilization are determined by the subtle balance among electronic, orbital, and lattice degrees of freedom.^1–4^

In this context, particularly promising is the use of quasi-zero dimensional systems, such as semiconductor quantum dots (QDs), which exhibit a high energy harvesting/conversion efficiency,^2,5^ and strong light emission enhancement.^6^ Despite their electronic structure is made of discrete levels similar to isolated atoms, QDs are generally grown in a solid-state environment, which is responsible for strong dephasing and decoherence effects detrimental for their optical, electronic, and thermal properties.^7–13^ In order to fully exploit their potential, it is therefore crucial to understand the interaction between the electronic and atomic degrees of freedom both within the dots and with the surrounding crystal lattice.

Ultrashort electron pulses with energy ranging from few tens to few hundreds of keV exhibit a high scattering cross-section and a de Broglie wavelength on the order of picometers, allowing one to achieve high spatiotemporal resolutions in diffraction, imaging, and spectroscopy.^14–18^ In particular, ultrafast electron diffraction (UED) is able to probe the dynamics of nanomaterials down to the atomic-scale,^16,19–22^ and has become the method of choice for the investigation of electron-phonon coupling,^23–27^ atomic motions,^28–35^ phase transitions,^36–38^ acoustic wave propagation,^39,40^ and bond dynamics in proteins.^41,42^

Here, we employ UED in the reflection geometry to investigate the lattice dynamics in GaAs quantum dots (QDs) grown on AlGaAs. An ultrafast optical excitation nearly resonant with the lowest electronic transition of the dots is adopted to create a confined exciton. The transient change of the diffraction pattern measured as a function of the delay time between the optical pump and the electron probe is then used to map the phonon dynamics both within the dots and in the substrate region surrounding them. We found that the excited electronic distribution efficiently couples to acoustic phonons through the deformation potential (DP) mechanism. In agreement with previous theoretical calculations,^43–45^ we interpret our observations as a result of a nonequilibrium phonon population composed of two parts: one localized within the dots, as detected from the Bragg reflections, and one traveling at the speed of sound in the surrounding region as a phonon wavepacket (WP), as retrieved from the dynamics of surface wave resonance (SWR) features.

## MATERIALS AND METHODS

**FIG. 1.**
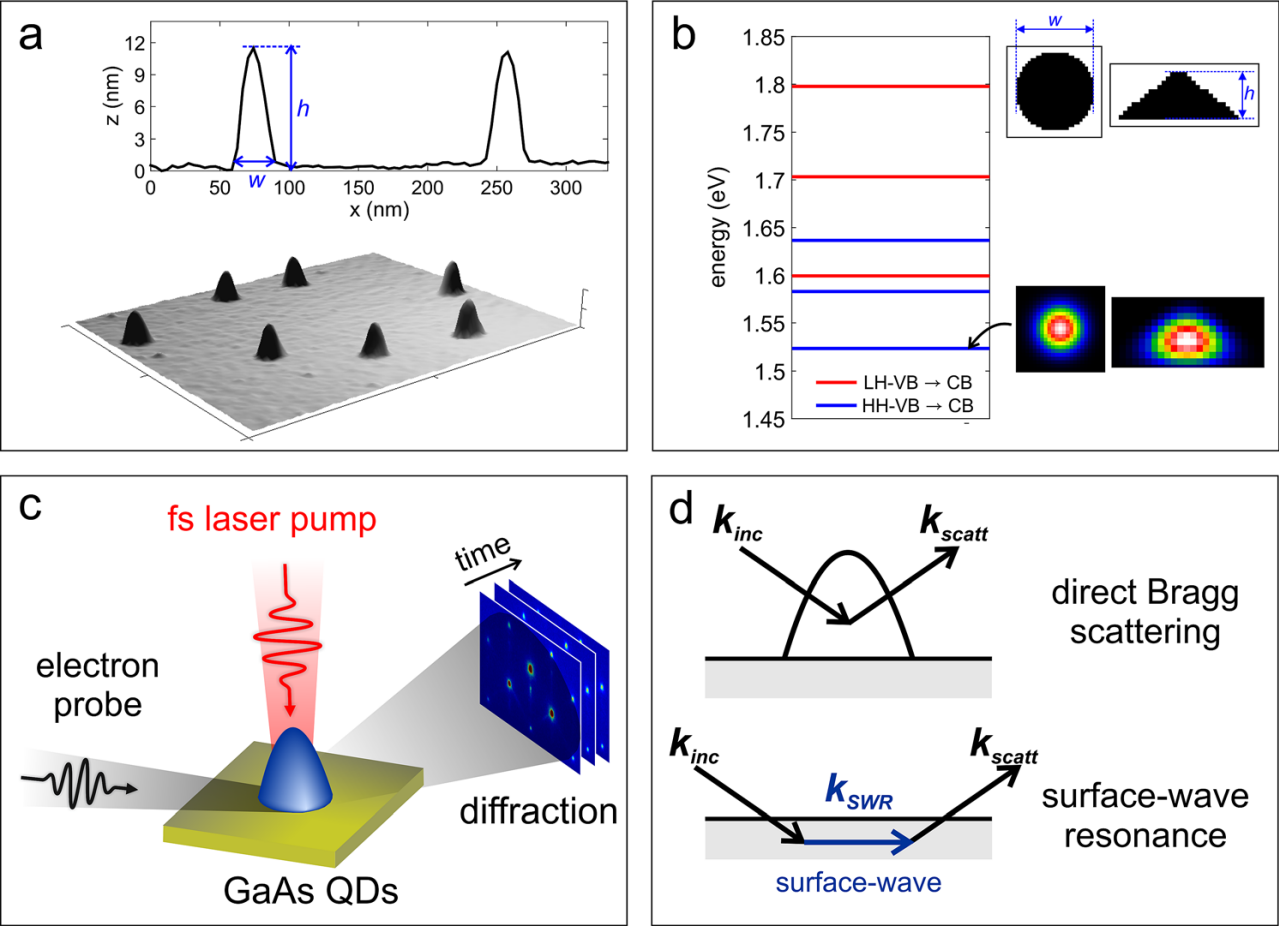
Quantum dots: morphology, electronic structure, and diffraction experiment. (a) Morphological characterization of the investigated quantum dots performed by atomic force microscopy (AFM). The average height is *h* = 11 nm and the average base width is *w* = 31 nm. (b) Calculated interband excitonic transitions for the investigated quantum dots using nextnano3 software package.^48^ The geometry used in the calculations and the electronic wavefunction for the lower transition at 1.52 eV are shown as insets. (c) Schematic representation of the ultrafast electron diffraction experiment: ultrashort electron pulses are focused on the sample in grazing incidence geometry; the dynamics is initiated by 120 fs laser pulses at 800 nm focused in the normal incidence; the diffracted electrons are then recorded in the stroboscopic mode at different delay times between the excitation laser and the electron pulse. (d) Schematic representation of diffraction geometry for Bragg scattering and surface wave resonances (SWR). In Bragg scattering, the incoming electron wave is directly coupled to an outgoing wave, whereas for SWR the incident beam is not directly scattered into an outgoing beam but couples with a beam nearly parallel to the surface before being emitted.

GaAs quantum dots without wetting layer were grown by droplet epitaxy (DE)^46^ on a 90-nm thick Al_0.3_Ga_0.7_As layer deposed on an n-type doped GaAs(001) substrate. The DE method consists of a two-step procedure. First, Ga droplets are created by the deposition of 0.25 monolayers (ML) of gallium at 200 °C with a flux of 0.05 ML/s. In order to prevent the formation of a wetting layer, 1 ML of Al_0.3_Ga was deposited on c(4 × 4) As-stabilized before the deposition of Ga. Then, a flux of As_4_ (equivalent pressure ∼ 5 × 10^−5 ^Torr) was used to irradiate the surface and crystallize the Ga droplets into GaAs dots, while the substrate temperature was reduced down to 150 °C. After the growth, the samples were capped with an amorphous As layer able to prevent the formation of native oxide due to air exposure during the following transfer to the UED setup. Prior to the experiments, the As capping layer was removed by annealing the samples *in situ* at 320 °C for 30 min, producing a clean and atomically flat surface. Morphological characterization of the dots has been performed by atomic force microscopy (AFM) and is reported in Fig. 1(a). The size and shape distributions are highly homogeneous, yielding dome-shaped dots with an average height *h* = 11 nm and an average base width *w* = 31 nm.

The electron band energetics of a GaAs/Al_0.3_Ga_0.7_As heterostructure induces a confinement of both electrons and holes within the dot. When the dot size is smaller than the exciton radius (∼12 nm for GaAs), quantum confinement effects become relevant, giving rise to a discrete energy spectrum and an increase of the lowest excitonic transition. Figure 1(b) displays the results of *k-dot-p* (or effective mass approximation) calculations^47,48^ for the interband transitions in the dot geometry studied in this work. The lowest transition is at 1.52 eV, which is nearly resonant with the adopted optical excitation at 800 nm.

The diffraction experiments have been conducted using an ultrafast electron diffraction setup at Caltech working in the reflection geometry, as schematically depicted in Fig. 1(c). Ultrashort electron pulses with energy per particle of 30 keV and sub-picosecond pulse duration are generated in a photoelectron gun (Kimball Physics, Inc.) after irradiation of a LaB_6_ photocathode with ultrashort UV laser pulses (λ = 266 nm). The electron beam has a transverse spot size of ∼100 *μ*m and is focused on the sample in a grazing incidence geometry (0.5°–2.5°). The sample is mounted on a 5-axis goniometer, allowing for simultaneous adjustment of the incidence (ϑ) and the azimuthal (ϕ) angles. The electron beam diffracted from the surface is recorded on a phosphor-screen/MCP/CCD assembly. The dynamics is initiated by 120 fs laser pulses at 800 nm (repetition rate of 1 kHz) with a fluence $f_{0}=4.6\;\text{mJ}/{\text{cm}}^{2}$ focused in the normal incidence on the sample surface. The velocity mismatch and the non-coaxial geometry between electrons and photons are responsible for a broadening of the temporal resolution. This effect is compensated for by tilting the wavefront of the optical pulse with respect to its propagation direction.^49^ The diffracted electrons are then recorded in the stroboscopic mode at different delay times between the excitation laser and the electron pulse.

## KINEMATICAL AND DYNAMICAL DIFFRACTION

**FIG. 2.**
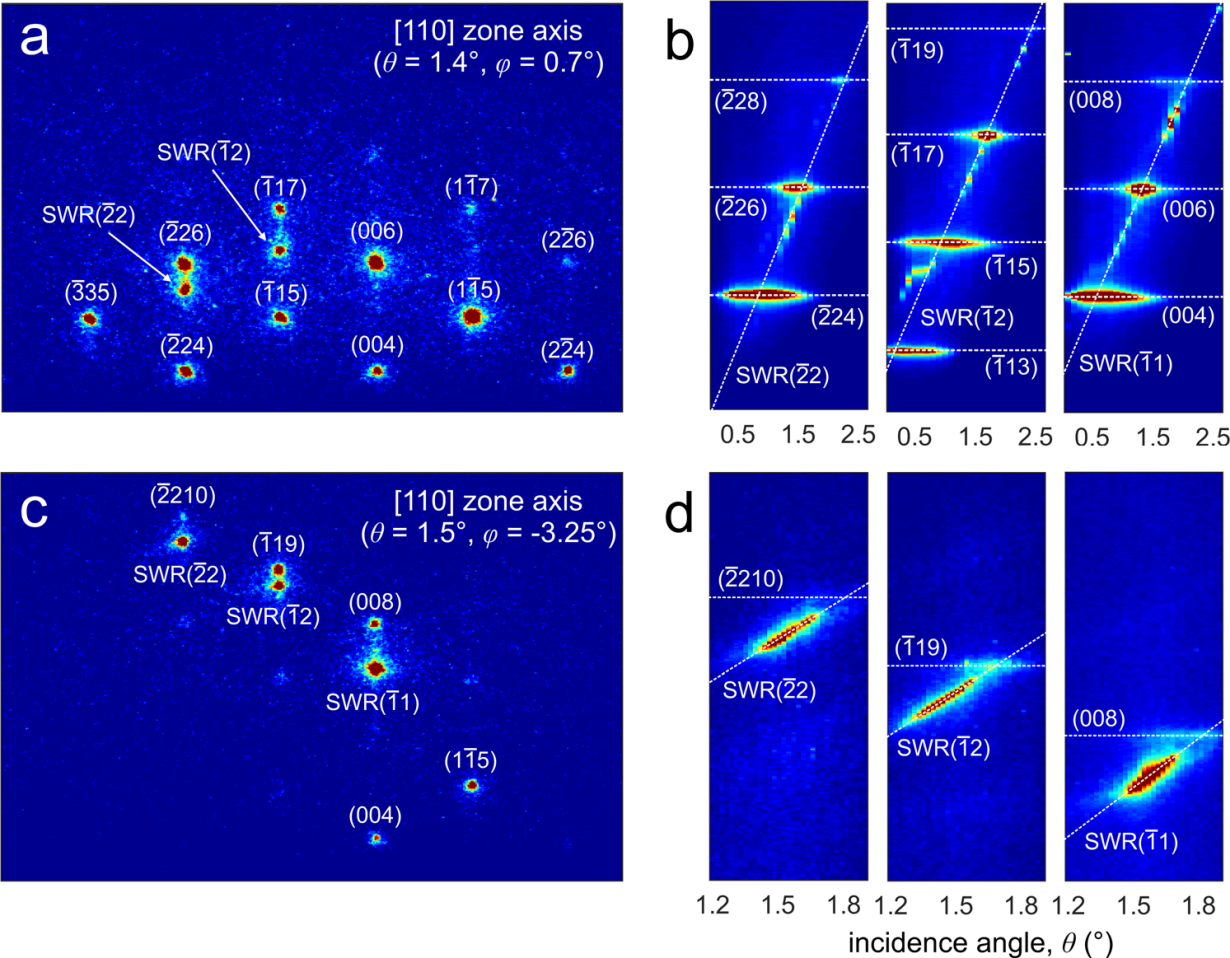
Diffraction from quantum dots: Bragg spots and surface wave resonances. (a) and (c) Static diffraction patterns taken with the electron beam propagating nearly along the [110] direction at two different azimuthal angles (ϕ=0.7° and ϕ=−3.25°, respectively). The assignment of the Bragg spots, which originate from the 3D morphology of the dots, and of the SWRs is done according to the characteristic zinc blende structure of GaAs. (b) and (d) Rocking curves (diffraction intensity vs incidence angle) measured for the two azimuthal angles used in (a) and (c). The SWRs linearly disperses with ϑ, whereas the Bragg spots show no dispersion.

Figures 2(a) and 2(c) show the static diffraction patterns taken with the electron beam propagating nearly along the [110] direction at two different azimuthal angles. The assignment of the Bragg spots, which originate from the 3D morphology of the dots, is done according to the characteristic chevron pattern of GaAs. In Bragg scattering, the incoming electron wave is directly coupled to an outgoing wave [see Fig. 1(d)], and the process can be described within the framework of the kinematical diffraction theory. For a weak inner potential, the scattering condition is

$$2d\;\text{sin}\;\vartheta =n\lambda_{e},$$ (1)

where $\lambda_{e}=0.69\times 10^{-11}\;m$ is the electron wavelength for electrons at 30 keV and *d* is the interplane separation. Surface wave resonance (SWR) features are also visible in the diffraction pattern. In this case, the incident beam is not directly scattered into an outgoing beam but couples with a beam nearly parallel to the surface before being emitted [see Fig. 1(d)]. The condition for the occurrence of surface wave resonances at a given ϑ and ϕ is obtained by imposing that the incident beam can diffract into a beam that is totally internally reflected (dynamical diffraction theory^29^). This results in

$$\vartheta^{2}=\left(k_{SWR}^{2}/k_{0}^{2}\right)-2\phi \left(k_{SWR}/k_{0}\right),$$ (2)

where $k_{SWR}=\left(2\pi /a\right)\sqrt{h_{m}^{2}+k_{m}^{2}}$ is the SWR wavevector (a is the in-plane lattice parameter, and $h_{m}$ and $k_{m}$ are the in-plane Miller indices) and $k_{0}=2\pi /\lambda_{e}$ is the electron wavevector. The expression in Eq. (2) shows that the SWR condition corresponds to an enhancement of the scattering intensity when a Kikuchi line crosses the integer-order beams (reciprocal lattice rods). This implies that the position in the diffraction pattern at which the SWR spot appears changes when changing the incidence angle. This is clearly visible in the rocking curves reported in Figs. 2(b) and 2(d), where the SWR linearly disperses with ϑ, whereas the Bragg spots show no dispersion.

It is worth noting that, because only ∼5% of the AlGaAs surface is occupied by the GaAs dots, the contribution to the SWR features of the multiple scattering events involving both dots and the surface is negligible. The strong dependence of the SWR resonance angles on the in-plane lattice parameter can thus allow to map the structural state of the material surface with high sensitivity.

## ULTRAFAST PHONON DYNAMICS

In the diffraction theory, the diffraction intensity, *I*, is proportional to the square modulus of the structure factor, F, that is, $I\propto {\left|F\right|}^{2}$. Because *F* is strongly dependent on the atomic displacement, u, the temporal behavior of the observed diffraction pattern is able to mirror the evolution of the lattice dynamics within the dots and in the region surrounding them. The involved motions are those of optical phonons, acoustic phonons, and incoherent thermal vibrations, which occur on well-defined and separate time scales.

**FIG. 3.**
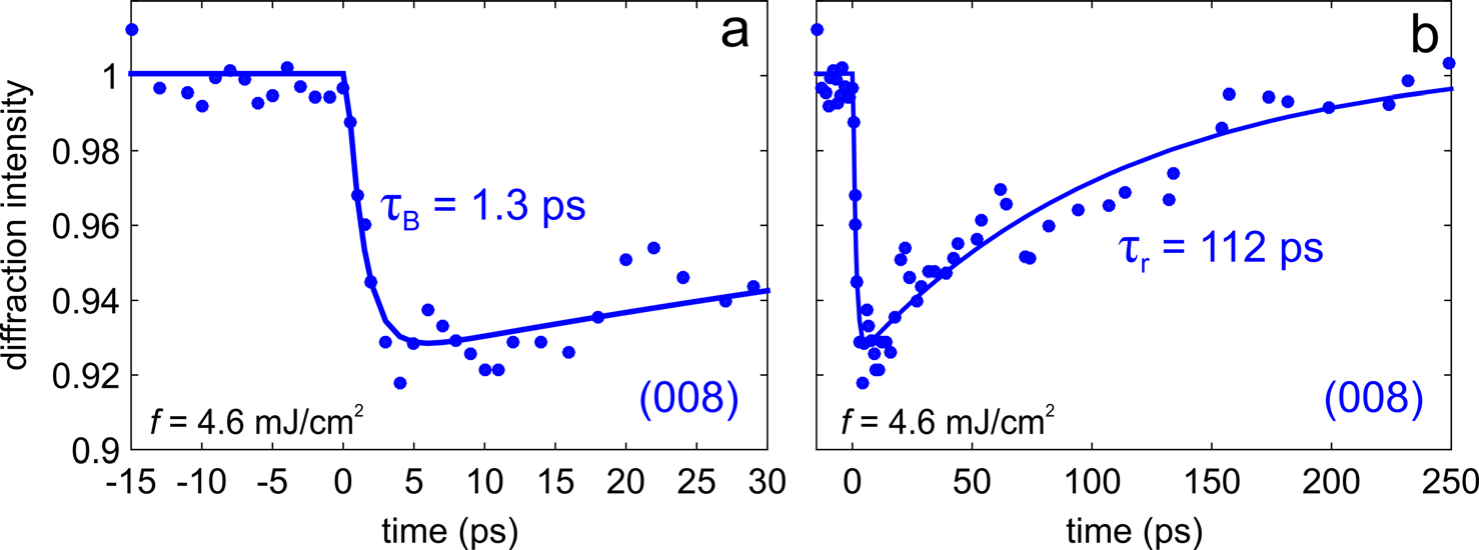
Ultrafast Bragg dynamics. The observed temporal change of the diffraction intensity for the (008) Bragg spot is shown at earlier (a) and later (b) delay times. The transient can be well described by a fast exponential decay with a time constant $\tau_{B}=1.3\;\text{ps}$ and a slower recovery to the equilibrium on a time scale $\tau_{r}=112\;\text{ps}$.

**FIG. 4.**
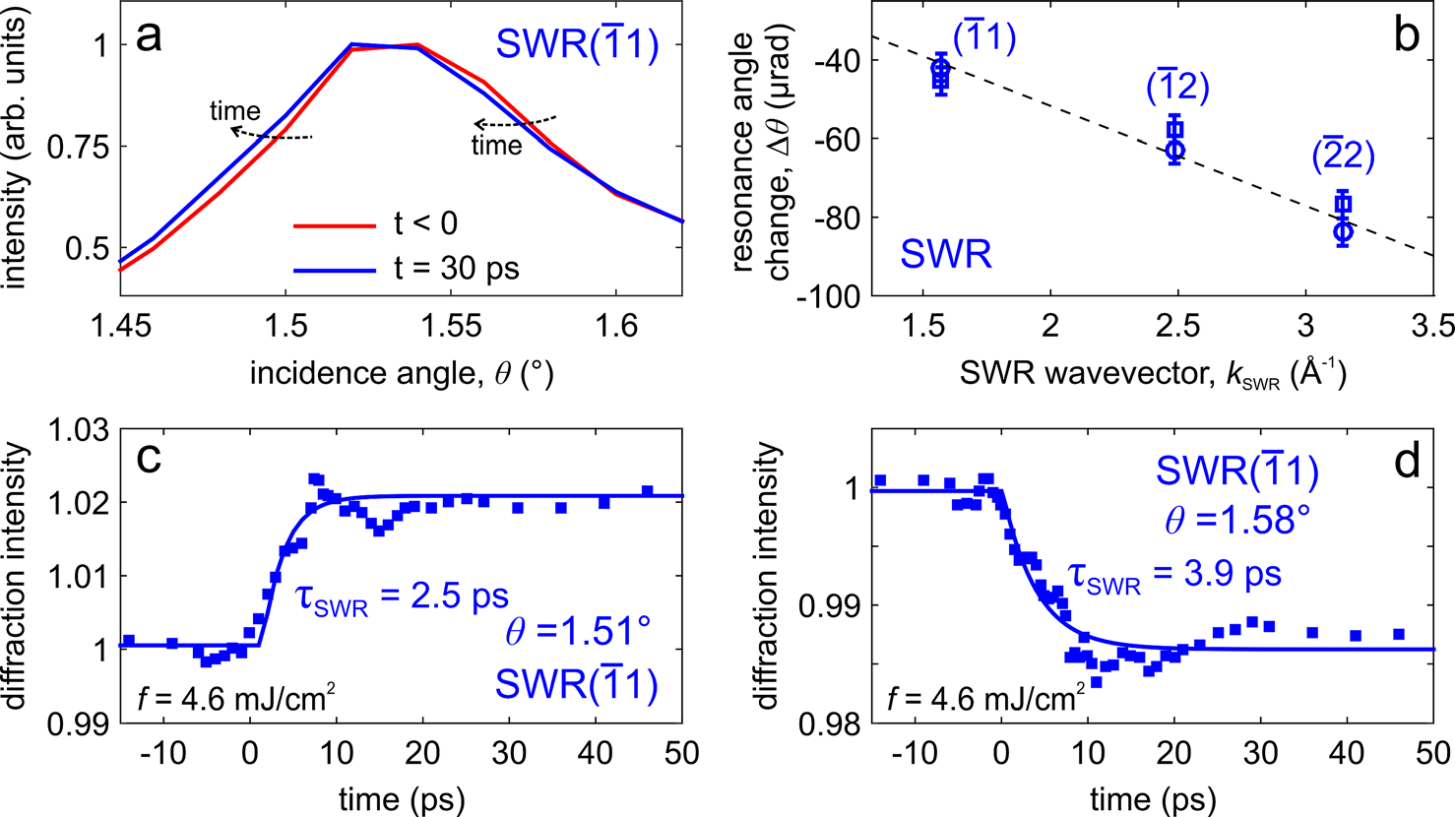
Ultrafast SWR dynamics. (a) Measured rocking curve for SWR $\left(\bar{1}1\right)$ at two different delay times: t = 0 (red line) and t = 30 ps (blue line). A clear shift of the resonance angle toward lower values is observed. (b) The shift of the SWR resonance angle is plotted as a function of the SWR wavevector for the observed features [SWR $\left(\bar{1}1\right)$, SWR $\left(\bar{1}2\right)$, and SWR $\left(\bar{2}2\right)]$. (c) and (d) Observed diffraction intensity transients measured for the SWR $\left(\bar{1}1\right)$ at incidence angles ϑ=1.51° (c) and ϑ=1.58° (d), which are, respectively, smaller and larger than the SWR resonance angle $\vartheta_{SWR}\approx 1.53{}^{\circ}$. From the measured transients, we estimated a time constant $\tau_{SWR}=2.5-4\;\text{ps}$ for the observed shift of the resonance angle.

In Fig. 3, we report the observed temporal change of the diffraction intensity for the (008) Bragg spot, which is related to the dynamics within the dots. The transient can be well described by a fast decay with a time constant $\tau_{B}=1.3\;\text{ps}$ and a slower recovery to the equilibrium on a time scale $\tau_{r}=112\;\text{ps}$. Particularly interesting is the lattice dynamics in the region surroundings the dots, which can be retrieved by monitoring the temporal behavior of the surface wave resonances (SWR). The results are shown in Fig. 4. Because of their peculiar dispersion relation as a function of the angle ϑ, we measured the full rocking curve at different delay times. When comparing the curves measured at t = 0 and at t = 30 ps [see Fig. 4(a)], a clear shift of the resonance angle toward lower values is observed, which proportionally increases (in the absolute value) for SWRs exhibiting a larger scattering vector [see Fig. 4(b)]. The shift of the resonance angle, $\vartheta_{SWR}$, creates a peculiar temporal evolution of the SWR intensity, $I_{SWR}$, for a fixed incidence angle. For ϑ smaller than $\vartheta_{SWR}$, $I_{SWR}$ increases with the delay time [see Fig. 4(c)], whereas for ϑ larger than $\vartheta_{SWR}$, $I_{SWR}$ decreases with time [see Fig. 4(d)]. From the measured transients, we estimated a time constant $\tau_{SWR}\approx 2.5-4\;\text{ps}$ for the observed shift of the resonance angle.

It is worth mentioning that for both Bragg spots and SWRs, we can exclude all the contributions to the observed dynamics related to field-induced deflection and to the influence of subsurface charge carrier distribution. First, because the optical excitation used in our work is 1.55 eV, which is larger than the band gap of ∼1.8 eV for the AlGaAs substrate, photoexcitation by the pump laser in the substrate is negligible and thus the influence of the subsurface charges as described in Ref. 50 can be ignored. Second, as described in Ref. 51 in the case of a reflection geometry, the field-induced deflection is independent of the diffraction angle ϑ, whereas the surface potential-induced shift of the diffraction peak decreases (in absolute value) for an increasing diffraction angle^52^ (non-reciprocal behavior). Both behaviors are not consistent with the measured SWR dynamics, where the variation of the SWR resonance angle, ${\Delta \vartheta }_{SWR}$, increases (in the absolute value) for an increasing diffraction angle (reciprocal behavior) as found for the $\left(\bar{1}1\right)$, $\left(\bar{1}2\right)$, and $\left(\bar{2}2\right)$ resonances [see Fig. 4(b)]. Third, the non-reciprocal behavior, excluding both field-induced deflection and refraction-induced shift, is also present for Bragg peaks. For the (00n) Bragg spots, we found that Δϑ scales linearly with the diffraction angle: Δϑ/ϑ is 1.9 × 10^−3^ for the (004), 2 × 10^−3^ for the (006), and 1.8 × 10^−3^ for the (008). Finally, the field-induced deflection and the refraction-induced shift evolve on a time scale of several tens of ps,^51,52^ which is considerably longer than the observed time constant for the measured SWR and Bragg spot dynamics. According to these reasons, the measured Bragg spots and SWR dynamics can only be associated to lattice changes induced by the photoexcitation of the quantum dots and the following structural evolution.

As mentioned above, the band gap of the AlGaAs substrate layer is ∼1.8 eV, and therefore the laser excitation at $E_{ph}=1.55\;\text{eV}$ is mainly localized within the dot, where the lowest electronic transition at ∼1.52 eV, associated to a confined bound electron-hole pair (exciton), is nearly resonantly excited by the photon pump. This creates a superposition of the ground state and the exciton state in the electronic part of the system, inducing a strong modification of the distribution of charge carriers within the dots. In these conditions, the deformation potential (DP) interaction, which relates the modification in energy of the electronic distribution to the deformation of lattice in the solid, is the predominant contribution for electron-phonon coupling, inducing a shift of the equilibrium position of the lattice ions and thus creating a local deformation. The stress associated with the DP mechanism (electronic pressure) can be obtained as

$$\sigma_{DP}=a_{def}N_{e},$$ (3)

where $a_{def}\approx 8.5\;\text{eV}$ is the deformation potential^53^ and $N_{e}$ is the number of excited carriers. The latter is given by

$$N_{e}=rf_{0}\frac{\left(1-R\right)}{\xi }\frac{E_{ph}-E_{gap}^{B}}{E_{ph}}\frac{1}{E_{ph}},$$ (4)

where R=0.3 is the reflection coefficient, r is the resonant absorption factor derived in Ref. 31, ξ=694 nm is the light penetration depth at 800 nm, and $E_{gap}^{B}=1.42\;\text{eV}$ is the GaAs bulk bandgap. By solving Eqs. (3) and (4), we estimated an electronic pressure within the dots of about 73 MPa, which is able to induce an average atomic displacement of ∼5 pm as calculated with the linear elasticity theory. This value is in good agreement with the experimental atomic displacement of ∼4.6 pm obtained from the intensity change observed for the (008) Bragg spot:

$$\langle u^{2}\rangle =\left(2/s_{\left(008\right)}^{2}\right)\text{ln}\left(I_{0}/I\right),$$ (5)

where $s_{\left(008\right)}=2\pi 8/a$ (a=5.65 Å) is the (008) scattering vector and $I_{0}/I=1/0.92$. This instantaneous electron-driven modification of the lattice ions triggers the excitation of optical and acoustic phonons forming a localized polaron within the dot. A polaron is an entangled electron-phonon quasi-particle and its wavefunction can be quantum mechanically represented by the coherent superposition of an electronic part and a phononic contribution. First principles calculations determined that acoustic phonons are excited on a time scale of about 1 ps from the laser excitation,^8,44^ which is similar to the time constant of 1.3 ps experimentally observed in the (008) transient. Because the dot height is smaller than the phonon inelastic mean free path ($\Lambda_{p}\approx 27\;\text{nm}$), acoustic phonons can ballistically propagate within the dot without suffering additional scattering. This process has been already addressed in detail in Ref. 31. Here, we only mention that the diffusive scattering at the GaAs/AlGaAs interface (heat dissipation) determines the time scale for the recovery toward the equilibrium state. In the framework of a thermal equilibrium model, the heat diffusion away from the dot is determined by the heat transport within the AlGaAs layer and therefore evolves on a time scale

$$\tau_{\text{heat}}\approx h_{\text{AlGaAs}}^{2}/\left(4D_{int}\right),$$ (6)

where $h_{\text{AlGaAs}}=90\;\text{nm}$ is the AlGaAs layer thickness and $D_{int}$ is the heat diffusion coefficient at the interface. The latter is given by

$$D_{int}=k_{int}/C_{\text{AlGaAs}},$$ (7)

where $k_{int}=\left(k_{\text{GaAs}}+k_{\text{AlGaAs}}\right)/2$ is the interface thermal conductivity [$k_{\text{GaAs}}=57.95\;W/\left(\text{mK}\right)$ and $k_{\text{AlGaAs}}=13.72\;W/\left(\text{mK}\right)$ are the thermal conductivities in GaAs and AlGaAs, respectively], and $C_{\text{AlGaAs}}=1.77{\times 10}^{6}\;J/\left(m^{3}K\right)$ is the AlGaAs heat capacity. By solving Eqs. (6) and (7), we can predict that heat dissipation occurs on a time scale of ∼100 ps, which is very close to the value of $\tau_{r}=112\;\text{ps}$ experimentally measured.

The incoherent diffusion of acoustic phonons across the GaAs/AlGaAs interface and within the AlGaAs layer cannot, however, explain the dynamics observed for the surface wave resonances. In fact, in the case of a simple thermal heating of the substrate, the time scale of the SWR transients should be determined by the heat transport from the dots within the surface region. The time scale for the surface heat transport can be calculated within a 2D diffusion model and depends on the lateral size of the dot, *w*, and on the diffusion coefficient in AlGaAs, $D_{\text{AlGaAs}}$:

$$\tau_{\text{Surf}-\text{heat}}\approx w^{2}/\left(4D_{\text{AlGaAs}}\right),$$ (8)

which turns out to be about 31 ps. This is contrary to our experimental observations, where a time constant of 2.5–4 ps is measured and can rather be interpreted according to the following scenario. It has been theoretically predicted^43–45^ that the deformation potential mechanism responsible for the excitation of acoustic phonons within the dots is associated to the buildup of a nonequilibrium phonon distribution consisting of two parts: a localized one that remains within the dots and reflects the polaronic nature of the excited state, as discussed above, and another part that, because of the spatial dispersion of the acoustic phonons, leaves the dot and propagates in the surrounding regions as a phonon wavepacket (WP) travelling at the speed of sound. In this scenario, the time necessary for the travelling wave to leave the dot and propagate in the surrounding substrate can be estimated as

$$\tau_{WP}=h/v_{A},$$ (9)

where h is the dot height and $v_{A}$ is the acoustic phonon speed. In our case, we have h=11 nm and $v_{A}=3.2-4.0\;km/s$ for the high-frequency acoustic phonons excited by the polaron decay, resulting in $\tau_{WP}=2.75-3.43\;\text{ps}$, which is in good agreement with the measured time constant of 2.5–4 ps for the observed SWR transients.

**FIG. 5.**
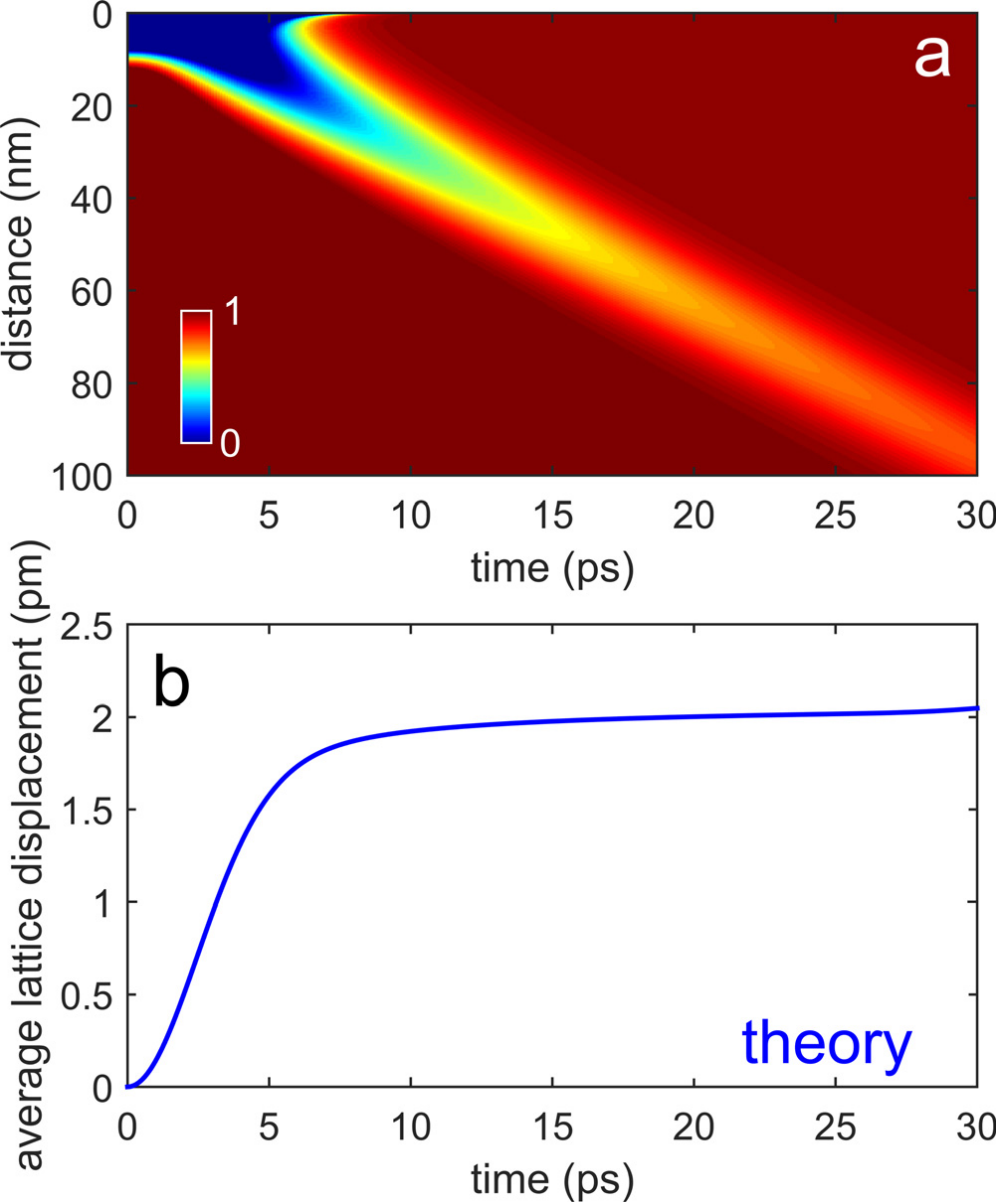
Lattice displacement calculations. (a) Normalized lattice displacement induced in the substrate as a function of the distance ***r*** from the dot and of the delay time since the photoexcitation, as due to acoustic phonons propagating away from the dot. The calculations are done adopting Eq. (9) in Ref. 43, with a confinement length defined by the dot height (*h* = 11 nm) and all the parameters provided in Ref. 44 for GaAs. (b) Spatial average of the calculated lattice displacement in the region around the dots to be compared with the SWR dynamics. The resulting curve shows a transient with a time constant of about 3–4 ps and a stationary value of $\left\langle u\right\rangle \approx 2.1\;\text{pm}$ for $t\gg h/v_{A}$.

When studying the lattice dynamics of a system of interest, the physical quantity used to quantitatively describe the process is the lattice displacement associated with acoustic phonons during their propagation. In a recent publication, Wigger *et al.*^43^ have calculated the expectation value of the lattice ion displacement, $\left\langle u\left(r,t\right)\right\rangle$, induced in the substrate region at a distance ***r*** and after a delay time *t*, as due to acoustic phonons propagating away from the dot. From their calculations, the travelling component appears as a Gaussian-shaped packet centered at $r=v_{A}t$ and decaying as ∼1/*r*. Adopting a confinement length as defined by the dot height (*h* = 11 nm) and the parameters provided in Ref. 44 for GaAs, the normalized average lattice displacement calculated from Eq. (9) in Ref. 43 is reported in Fig. 5(a), clearly showing the space-time propagation. Because in our case the electron spot size is considerably larger than the separation between the dots, a proper comparison with the measured SWR dynamics can be obtained only when spatially averaging the calculated lattice displacement in the region around the dots. The resulting curve is shown in Fig. 5(b) and shows a transient with a time constant of about 3–4 ps with a stationary value of $\left\langle u\right\rangle \approx 2.1\;\text{pm}$ for $t\gg h/v_{A}$. Because the phonon wave packet has limited spatial and temporal extensions, the calculated value represents the largest possible displacement induced within the lattice, which is achieved on a time scale determined by $\tau_{WP}$. The theoretical atomic change has to be compared with the experimentally measured in-plane lattice change, Δa, obtained from the observed shift of the SWR resonance angle. Differentiating Eq. (2) for a constant ϕ gives

$$\Delta a/a=-\Delta \vartheta /\vartheta \left[\frac{\left(k_{SWR}^{2}/k_{0}^{2}\right)-2\phi \left(k_{SWR}/k_{0}\right)}{\left(k_{SWR}^{2}/k_{0}^{2}\right)-\phi \left(k_{SWR}/k_{0}\right)}\right]$$ (10)

and using the results shown in Fig. 4(b), we find that Δa≈1 pm, which is in substantial agreement with the value of ⟨u⟩≈2.1 pm calculated above. We can therefore interpret the observed SWR dynamics as the fingerprint of the lattice change associated to the phonon emission from optically excited quantum dots.

An important part of the narrative is the generation process of the emitted phonons. Two mechanisms can take place: the first is related to the phonon emission as driven by the excess energy released during the formation of the polaron, while the second one is associated to energy relaxations mediated by real electronic transitions. In our case, the laser excitation at 800 nm nearly resonantly excites the lowest excitonic transition with no other states available at lower energies. Therefore, we believe that the first scenario applies, as also predicted by theoretical calculations.^45^ The excess energy is about 30 meV, which is enough to excite acoustic phonons within a large range of the Brillouin zone. This is essential for the buildup of a phonon wavepacket, which is composed by the superposition of phonons with a large number of different wavevectors where the lower cutoff is determined by the dot size ($q_{c}=2\pi /h\approx 5.7\times 10^{8}\;m^{-1}$).

Finally, we note that phonon emission from quantum dots has been experimentally probed on a nanosecond time scale and with millimeter resolution using bolometric detection techniques.^54,55^ Here, using the ultrafast electron diffraction approach, we have been able to map both the phonon localization within the dots and the phonon generation from them on the spatial and temporal scales of their occurrence, i.e., atomic and sub-picosecond, respectively.

## CONCLUSIONS

The theoretical description of the exciton-phonon coupling in semiconductor quantum dots involves the buildup of a strongly nonequilibrium phonon population composed of two parts: one localized within the dot and associated to the polaronic nature of the excited state, and another leaving the dot as a phonon wavepacket travelling at the speed of sound.^43–45^ To experimentally probe this behavior, we have induced a nearly resonant excitation of the lowest excitonic transition in GaAs quantum dots grown on AlGaAs using femtosecond light pulses at 800 nm. By means of ultrafast electron diffraction, we have monitored the lattice dynamics both within the dots, by observing the transient behavior of their Bragg reflections, and in the substrate region, by observing the dynamics of surface wave resonance (SWR) features. We found that the coupling between the confined exciton and the population of acoustic phonons is mainly mediated by the deformation potential mechanism. Our observations are fully consistent with the theory and experimentally confirm—with atomic sensitivity and sub-picosecond resolution—the generation of phonons from zero-dimensional systems. Because quantum dots of III–V materials are generally used in opto-electronic devices, these results demonstrate that III–V photonic-phononic emitters for communication technology are fundamentally feasible, as recently demonstrated for silicon nanostructures.^56^
